# A Visual Metaphor Describing Neural Dynamics in Schizophrenia

**DOI:** 10.1371/journal.pone.0002577

**Published:** 2008-07-09

**Authors:** Nico J. M. van Beveren, Lieuwe de Haan

**Affiliations:** 1 Erasmus University Medical Center, Department of Psychiatry, Rotterdam, The Netherlands; 2 Department of Psychiatry, Academic Medical Center/de Meren, Amsterdam, The Netherlands; Chiba University Center for Forensic Mental Health, Japan

## Abstract

**Background:**

In many scientific disciplines the use of a metaphor as an heuristic aid is not uncommon. A well known example in somatic medicine is the ‘defense army metaphor’ used to characterize the immune system. In fact, probably a large part of the everyday work of doctors consists of ‘translating’ scientific and clinical information (i.e. causes of disease, percentage of succes versus risk of side-effects) into information tailored to the needs and capacities of the individual patient. The ability to do so in an effective way is at least partly what makes a clinician a good communicator. Schizophrenia is a severe psychiatric disorder which affects approximately 1% of the population. Over the last two decades a large amount of molecular-biological, imaging and genetic data have been accumulated regarding the biological underpinnings of schizophrenia. However, it remains difficult to understand how the characteristic symptoms of schizophrenia such as hallucinations and delusions are related to disturbances on the molecular-biological level. In general, psychiatry seems to lack a conceptual framework with sufficient explanatory power to link the mental- and molecular-biological domains.

**Methodology/Principal Findings:**

Here, we present an essay-like study in which we propose to use visualized concepts stemming from the theory on dynamical complex systems as a ‘visual metaphor’ to bridge the mental- and molecular-biological domains in schizophrenia. We first describe a computer model of neural information processing; we show how the information processing in this model can be visualized, using concepts from the theory on complex systems. We then describe two computer models which have been used to investigate the primary theory on schizophrenia, the neurodevelopmental model, and show how disturbed information processing in these two computer models can be presented in terms of the visual metaphor previously described. Finally, we describe the effects of dopamine neuromodulation, of which disturbances have been frequently described in schizophrenia, in terms of the same visualized metaphor.

**Conclusions/Significance:**

The conceptual framework and metaphor described offers a heuristic tool to understand the relationship between the mental- and molecular-biological domains in an intuitive way. The concepts we present may serve to facilitate communication between researchers, clinicians and patients.

## Introduction

Schizophrenia is a severe psychiatric disorder, characterized by the emergence at adolescence of psychotic phenomena: hallucinations, delusions and bizarre behavior. The neurodevelopmental hypothesis, which proposes a leading role for early aberrant brain development on which normal and/or abnormal brain maturation is superimposed has become the dominant paradigm for understanding the development of schizophrenia. The neurodevelopmental theory is usually underscored by a large amount of molecular-biological, imaging and genetic data which have been accumulated over the last two decades. Taken together, these findings point to reduced neuronal connectivity and synaptic stability. Psychotic symptoms are considered to be emergent properties on the psychological and behavioral level of the aberrantly developed neural system which start when during brain development a critical threshold is passed.

However, it remains difficult to understand how psychotic symptoms are related to disturbances on the molecular-biological level. Kapur [1] described this as: “doctor-patient interaction proceeds mainly at a ‘mind’ or ‘behavioral’ level of description. On the other hand, the preeminent theories regarding psychosis (…) are mainly neurobiological”. We think this is a major problem in contemporary psychiatry because it impedes researchers to convey findings to patients, clinicians, and the general community. The problem is that psychiatry as a science seems to lack a coherent system of terms linking the mental and molecular-biological domains [2].

In somatic medicine the use of some kind of metaphor to bridge the biological and phenomenological domains is not uncommon. For instance, the immune system is characterized by the defense-army metaphor. Though the immune system is notoriously difficult on a molecular level, and the specific molecular phenomena which happen during, say, an HIV infection, may be only within the grasp of experts, the defense-army metaphor functions as a bridge between lay and professionals and facilitates understanding in an intuitive way.

In this article we attempt to outline a heuristic framework that could provide a basis for uniting clinical phenomena and neurobiological theories. We will introduce what we have coined a ‘visual metaphor’ which is supposed to bridge the mental and neural domains. To this end we will combine two fields of research, namely the study of the behavior of complex systems and computer models of schizophrenia.

During the last two decades a large body of literature has appeared concerning the study of complex systems and its associated theoretical framework ‘complexity theory’ [3], [4]. Complex systems consist of a set of simple elements which interact with each other and the environment and change in time as result of these mutual interaction. In psychiatry and the life sciences the study of complex systems has been identified as a potential source of new ideas and viewpoints [5]–[13] as the brain can also be seen as a complex system [14]. I.e. Peled [15], [16] suggested to use concepts from systems theory to form an innovative diagnostic framework

The neurodevelopmental theory of schizophrenia has been supported by computer simulations of neural information processing. Hoffman and McGlashan reported a body of research on schizophrenia [17]–[20] resulting in a pathophysiological model coined Developmentally Reduced Synaptic Connectivity [21]. This model specifically posits that schizophrenia arises from critically reduced synaptic connectivity as a result of developmental disturbances of synaptogenesis and supports the neurodevelopmental theory.

Both research efforts depend greatly on computer models and complicated mathematical ideas which are unfamiliar to most psychiatrists. However, the ideas developed in both fields have great heuristic value. In this paper we will try to visualize findings using concepts from the behavior of complex systems.

This article will first briefly introduce some basic principles of computer models of mental processes, so-called Artificial Neural Networks (ANN). A conceptual framework which is related to the functioning of ANN is described and we show how this conceptual framework can be visualized. This is the visual metaphor we seek to describe. Subsequently, we describe how implementing developmental disturbances in ANN give rise to pathological phenomena, what their relationship is with ‘real-life’ pathology and how these phenomena can be understood in terms of the visual metaphor we introduced. Finally, we will show how psychotic symptoms originating from dopamine disturbances can be understood in terms of this visual metaphor.

## Results

### Introducing the metaphor (1): artificial neural networks and attractors

There is an extensive body of literature on the principles of ANN [22]–[24]. We describe a certain class of ANN, the *attractor neural network* [23]–[26].

**Figure 1:**
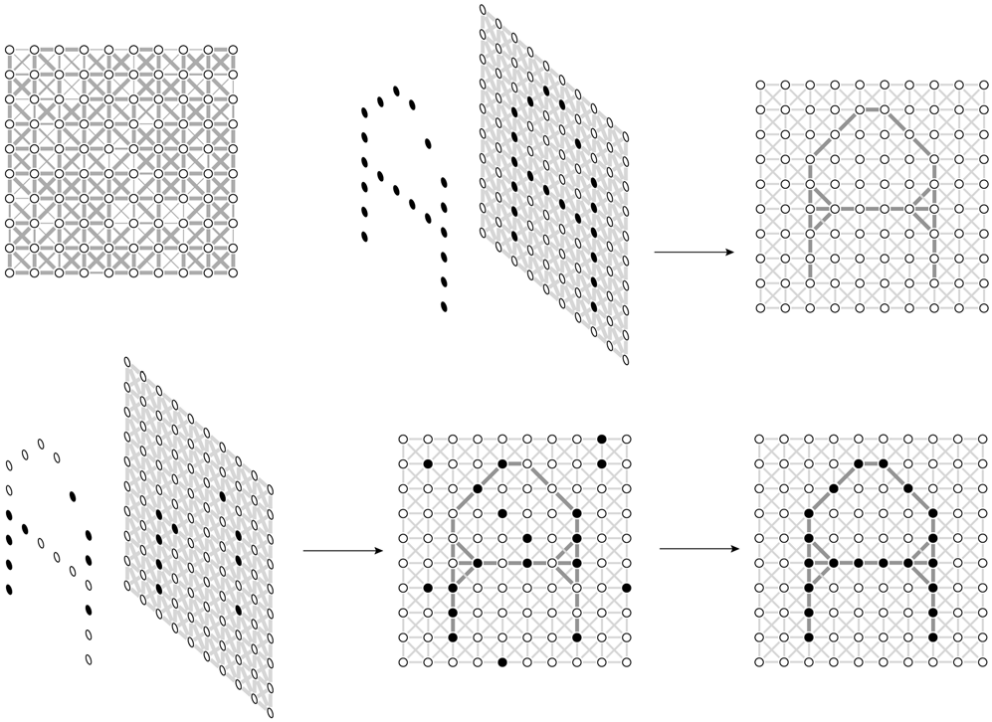
Learning an attractor network to recognize patterns. Figure 1, top, (left): a network with 100 units with connections of random strength and with no specific pattern learned (for clarity only neighbouring connections are shown). Figure 1, top (middle and right): learning pattern ‘A’. Top (middle): presenting ‘A’; top (right): connections between units related to ‘A’ are strengthened. Figure 1, bottom, (left): presenting part of ‘A’. Bottom (right): the network has succeeded in retrieving ‘A’

Figure 1 shows a network consisting of 100 artificial neurons (AN's) (figure 1, top, left). Each AN is connected with every other AN by means of connections of random strength (For clarity, only connections between neighboring AN's are shown). Each AN receives input from each of the 99 other AN's. Each AN can be active (‘1’) or inactive (‘0’ or ‘−1’). For example, the influence of each set of 99 AN's on the remaining one is given by the sum of the products of the number representing active or inactive and the strength of the corresponding connection. The remaining AN is itself active or inactive depending on whether the sum exceeds a certain threshold value or not. In this way, all the AN's continuously and mutually influence each other making this ANN a dynamic system. The functioning of this network can be simulated with a computer.

It is possible to present this ANN a certain pattern, for instance, a pattern that symbolizes the letter ‘A’ (figure 1, top, middle). The network is constructed such that connections between AN's which are simultaneously active become stronger; connections which are not simultaneously active become weaker.

After pattern ‘A’ has been presented, this mechanism will have strengthened the connections in the ANN in such a way that pattern ‘A’ has become imprinted in the connections between the AN's (figure 1, top, right). Pattern ‘A’ has now become an *attractor* of the network dynamics: the network has learned ‘A’ (‘learning can be regarded as collecting new attractors’ [19]. An attractor implies a preferent state of a dynamic system.

What this means is shown in figure 1 (bottom, left). In the left-hand side of the figure, some part of ‘A’ is presented. The activity of the ANN is continuously updated (figure 1, bottom, middle). The dynamic behavior of the network will finally be attracted towards pattern ‘A’, the attractor of the network (igure 1, bottom, right). The ANN is now said to have recognized the ‘A’ pattern. An ANN can have several attractors.

### Introducing the metaphor (2): visualizing the behavior of neural systems as a trajectory through state space

The behavior of systems like an ANN can be described with so-called ‘state space’ representations and from this concept we derive the ‘visual metaphor’ we like to introduce.

Mathematically, all the states in which the ANN can exist can be represented by a multidimensional ‘space’. The basic idea can be introduced by considering one neuron with two possible states, ‘firing’ (‘1’) and ‘non-firing’ (‘0’). These two states can be graphically depicted as two points. Over time, the dynamical behavior of this one-neuron ‘system’ can be represented by a *trajectory* that jumps between the two possible states. Similarly, a two-neuron system can exist in four different states and performs a trajectory that moves between these four points in a two-dimensional space; hence the term ‘state space’.

From a mathematical point of view, there is no difference with respect to the situation describing systems consisting of N neurons. They describe a trajectory through a N-dimensional space. Unfortunately, this situation cannot be visualized.

**Figure 2:**
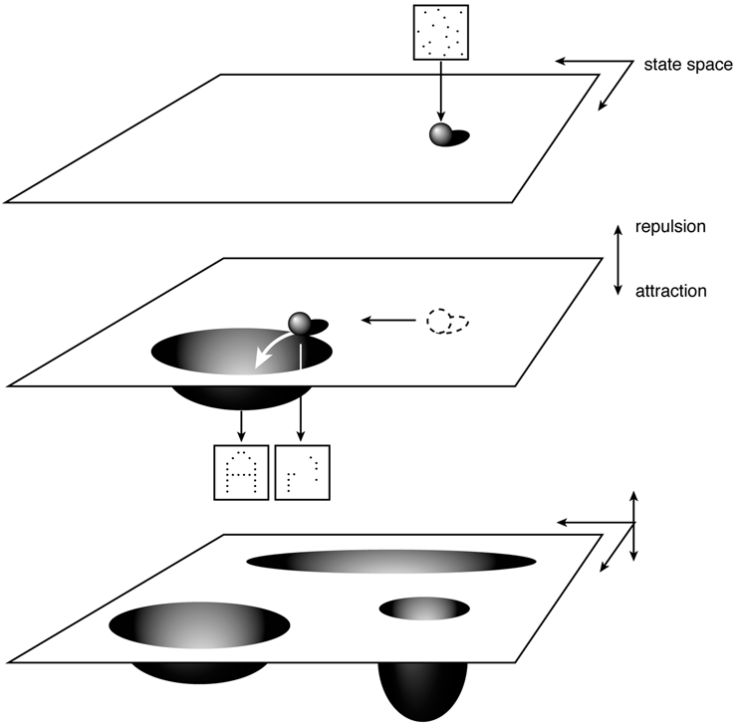
Dynamical behavior of a network as a trajectory in state space guided by attractors (for clarity repulsors are not shown). Figure 2, top: different states of a network represented by points in a plane: the state space. A random situation is shown. Figure 2, center: an attractor representing ‘A’ symbolised as a dent in state space. Changes in the state of the network can be regarded as a trajectory through state space. Figure 2, bottom: attractors with variably sized basins (representing the influence of the attractor) and variable depths (representing the preference of the system to stay in the attractor).

We propose to simplify this high-dimensional situation to the depiction of a plane with the different states as points in the plane (see figure 2, top; see also Globus and Arpaia [8] and Beer [13]. In fact we performed some kind of intuitive principal component analysis by assuming that relevant variance occurs in limited directions [13].

This plane represents the collection of states in which the system can exist. (Throughout we should keep in mind that in fact we are dealing with a multi-dimensional space which cannot be graphically shown).

An attractor (a learned pattern) can be represented as a dent in this plane (figure 2, center). The magnitude of the attractor, commonly described as the *basin of attraction*, represents the influence of the attractor over its surroundings. In psychological terms, it represents how much of a stored memory has to be ‘fed into’ the neural system in order for the ANN to perform an associative recollection of the memory. Changing from one state to another can be conceptualized as the movement of a ball over the surface [27]. In time, the system ‘moves over the plane’: it takes different states and by doing so performs a trajectory through the state space. With this conceptual framework we have a visual tool to aid our understanding of the functioning of the ANN. Because of its analogy with the movement of a ball over a landscape we have coined it a ‘visual metaphor’.

Presenting a pattern to an ANN can be likened to moving a ball over the surface and letting it loose. The system will then seek some state guided by the attractors which dominate the systems' behavior and will settle in one of its attractors (figure 2, center). A situation in which several attractors are present is shown in figure 2 (bottom). Throughout, we must keep in mind that this visual representation is a metaphor for what are essentially abstract mathematical concepts.

For a proper understanding, one has to imagine the dynamic aspects of the whole process. So, the state of the ANN is continuously moving through the space of its possible states guided by external influences and attractors with the ANN simultaneously creating new attractors which can be regarded as memory traces [15]. Moreover, in this kind of system, small changes in the state of the system can result in major changes in the final state of the system. These changes are known as *phase transitions* and appear when the system is near bifurcation points: unstable states in which the system chooses between one or another final state to evolve to. Such sudden changes in the state of the system can be imagined as popcorn, ‘popping’ from one attractor to another [17].

### Using the metaphor (1): the neurodevelopmental theory, ANN and psychopathology

Efforts to simulate schizophrenic pathology using ANN assumed that schizophrenia arises from reductions in connectivity between brain regions as a result of developmental disturbances during synaptogenesis [28], [29]. A first effort used the 10×10 ANN described earlier. After learning this ANN a number of symbols, a pruning rule was imposed on the ANN, removing weak connections [15]. However, at higher levels of pruning, the ANN demonstrated pathology. One of the findings was that parts of the network would tend to re-create a pattern of activation that did not correspond to any particular stored memory. It was hypothesized that autonomous activation of cortical areas arising independent of activity in other areas would be subjectively experienced as hallucinations or the experience that one's thoughts are being controlled by outside forces.

**Figure 3:**
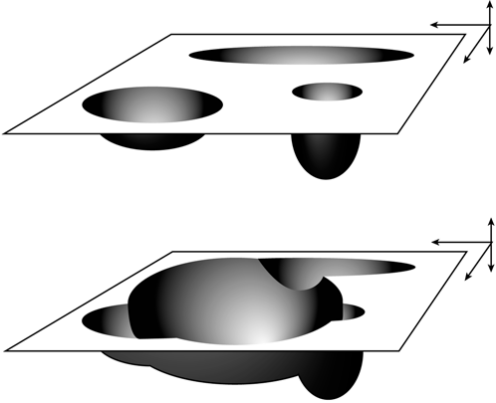
Pathology shown as disturbances in state space (see text). Figure 3, top: ‘normally’ sized attractors (cf figure 2, bottom). Figure 3, bottom: aberrant neurodevelopmental processes (i.e. excessive pruning) give rise to a spurious attractor: situations and concepts become malclassified (interpreted after 17). This may lead to the subjective experience of delusions or hallucinations.

We can visualize this situation using the introduced metaphor (see figure 3, bottom).

Excessive pruning has created a so-called ‘parasitic’ attractor. Input will not bring the ANN into the regions in state space which correspond to a previously learned symbol but to a newly emerged attractor. The re-creation of the pattern can be interpreted as autonomous activation of the ANN. In terms of dynamical behavior the pathologically configured ANN is performing an inappropriate trajectory through its state space. (Through a region of its state space not corresponding to any previously learned pattern, hence ‘inappropriate’).

Later studies involved a more complex ANN (a back-propagation network with a recurrent layer) focusing on developmentally disturbed speech processing as a source of auditory hallucinations [20], [30]. Normal human speech is a complex task given the high level of acoustic ambiguity. Normal perception of a word depends not only on acoustic input corresponding to the word itself but also on previously perceived words and intrinsic knowledge of how words are sequenced into larger messages. The process involves verbal working memory that uses expectations based on prior words and phrases.

This ANN used featured a verbal working memory with linguistic expectations built up from prior exposure to a training set of grammatical correct sentences. The training set consisted of sentences like “Jane kiss girl” or “Cop chase man”. The ANN was programmed to process degraded input signals into identifiable words. The ANN was posited to ‘hallucinate’ when it recognized words during periods of input silence.

Pruning of connections initially improved detection rates of phonetically degraded words. Higher levels of pruning however were associated with progressive impairment in word recognition and the emergence of hallucinations.

**Figure 4:**
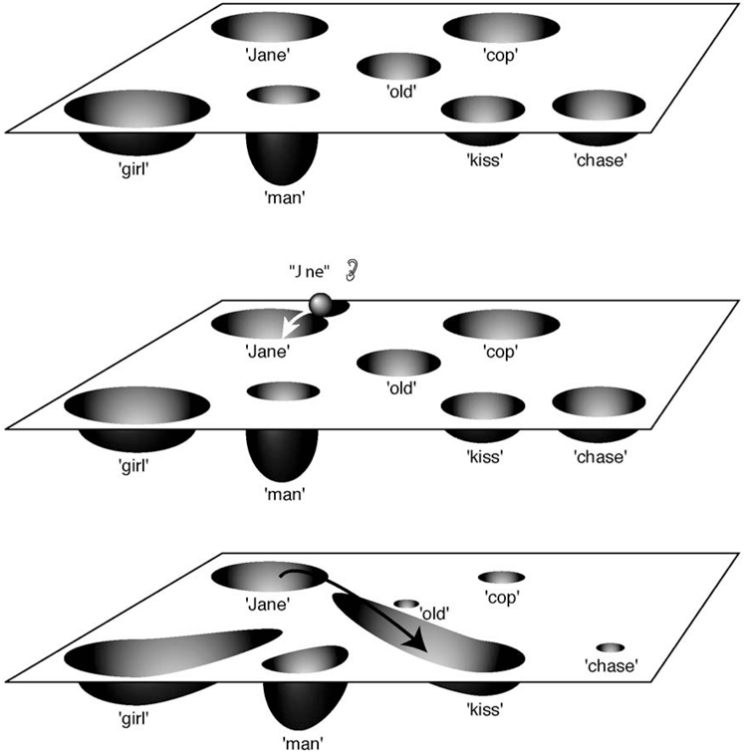
Dynamical aspects of the ‘attractor landscape’ (see also text). Figure 4, top: meaningful acoustical signals have created an ‘attractor landscape’. Figure 4, center: recognizing a (degraded) signal, ic ‘Jane’. Figure 4, bottom: after recognizing ‘Jane’ the basins of attraction of concepts associated with ‘Jane’ (acoustical signals, likely to follow ‘Jane’) temporarily increase, facilitating the capacity to recognize subsequent input (i.e. the most likely words to follow Jane).

Turning to the visual metaphor to describe what is happening (see figure 4) we should conceptualize the state space and its ‘attractor landscape’ as to a certain extent dynamical itself.

Figure 4 (top) depicts the starting situation in which linguistic concepts are depicted as attractors. The basins of attraction influence the trajectory through the state space as we have described for the simple ANN.

Figure 4 (center) shows the system ‘hearing’ a degraded acoustic stimulus (‘J?ne’). Due to the attractor dynamics (these depent on the basin of attraction of ‘Jane’ as well as the state the system is in) the network might succeed in recognizing ‘J?ne’ as ‘Jane’.

Perceiving a word (like “Jane”) will change basins of attraction (depending on prior expectations, i.e. the training set of sentences), and as a consequence the most likely trajectories that subsequently will be followed. Thus perceiving “Jane” will enlarge the basin of attraction of “kiss” and other words the ANN has learned to associate with “Jane” and at the same time shrink the basin of attraction of concepts associated with, for instance, “cop”. This situation is shown in figure 4 (bottom).

As a result, after perceiving “Jane”, the most likely trajectories will lead to regions of state space associated with “Jane” (like “kiss” or whatever other words the ANN due to its prior expectations expects to follow “Jane”).

The pruning process facilitates this process by improving the capacity to recognize degraded input, probably by enlarging basins of attraction and facilitating common trajectories (like “Jane” → “kiss” → “girl”). However, the drawback is that after a certain point recognizing becomes ‘too good’, and the system starts off to follow trajectories without any input: the system starts ‘hallucinating’.

### Using the metaphor (2): dopamine and schizophrenia

There is considerable evidence suggesting a role for dopamine involvement in schizophrenia, particularly motivated by the efficacy of antipsychotic medication, which derive their therapeutic effects from dopamine D2 antagonism [31]. Usually, research focusing on the role of dopamine in schizophrenia adresses two aspects of dopamine neuromodulation, namely (1) the role of exess dopaminergic stimulation in delusions, and (2) the role of low levels of prefrontal dopamine in working memory. The metaphor we present should be able to capture aspects of these roles of dopamine in schizophrenia.

### General aspects of the role of dopamine in neural system dynamics

An increasing body of research suggests that dopamine is a modulator of the signal-to-noise ratio of the neural system. One of the first to suggest this role were Servan-Schreiber et al [32]. In the metaphor presented, a crucial role is played by the attractor concept. The way in which information is processed is influenced by the shape and depth of the attractors and by the ease in which the system can settle into an attractor and perform transitions from one attractor into another. Changing the signal-to-noise ratio, in terms of ‘information processing with attractors’, comes down to changing the depth of the attractors and changing the magnitude of the basins of attraction.

**Figure 5:**
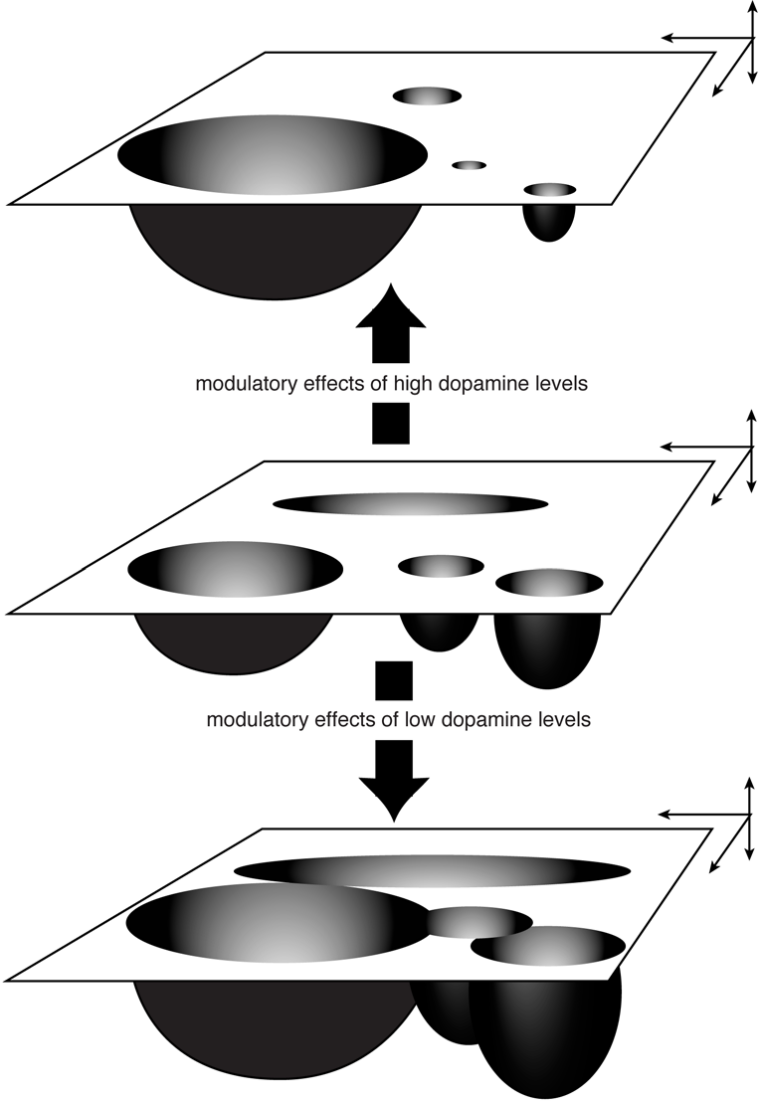
Modulatory effects of dopamine shown as changes in state space (interpreted after 30)(see also text). Figure 5, top: increasing levels of dopamine augment the signal-to-noise ratio: the basin of the attractor associated with the most salient signal increases while the basins of attraction of the other attractors decrease. Note also the increase respectively decrease of the depth of the attractors reflecting a increased respectively decreased tendency of the system to stay in the attractor. Figure 5, bottom: decreasing levels of dopamine reduce the signal-to-noise ratio: the basins of attraction and the depth of the attractors become more similar resulting in an enhanced tendency of the system to make transitions from one attractor to another.

Low levels of dopamine are associated with a low signal-to-noise ratio and in attractors terms this is equivalent to shallow attractors with large basins of attractions. In this situation frequent transitions from one attractor to another can take place (see figure 5, bottom). Servan-Schreiber at al [32] suggested that this is the psychological equivalent of relatively unfocused, associative thinking.

High levels of dopamine are associated with a large signal-to-noise ratio and this comes down to the existence of a limited number of deep atractors with relatively large basins of attraction which dominate the information processing of the neural system (figure 5, top). Such a situation will be present in a situation of focused attention.

### Excess dopaminergic stimulation in delusions

Recently, Kapur (1) presented an heuristic framework on the role of dopamine in delusions. In Kapur's model a central role of dopamine is to mediate the salience of environmental events. A (striatal) hyperdopaminergic state would lead, at a psychological level, to the aberrant assignment of salience to one's experience. Aberrant, because salience is assigned to experiences which (in fact) have none or only minor salience. Subsequently, delusions are the cognitive effort to make sense of aberrantly salient experiences.

Kapur (1) provides a psychological description of the role of dopamine. Within the framework we presented (based on ‘information-processing with attractors’) an abormal hyperdopaminergic state is equivalent to the emergence of an attractor with a large basin of attraction which dominates the striatal information processing of the neural system. This dominance translates itself in ‘aberrant salience’. This is the situation depicted in figure 5 (top).

### Low prefrontal dopamine in working memory

Whereas hyperdopaminergic states in the striatum have been associated with delusions, reduced dopaminergic activity in the prefronal cortex has been associated with disorders of working memory [33], [34]. Usually it is assumed that the prefrontal cortex is responsible for representing and maintaining task-relevant information, an idea related to the ideas of Goldman-Rakic concerning the role of the prefrontal cortex in working memory. Dopamine is involved in maintaining task-relevant information in the prefrontal cortex [35].

Schizophrenia patients show several deficits in psychological tasks, specifically in tasks that place a demand on the active maintenance of internal representations of the context of the task. Cohen & Servan-Schreiber [35] concluded that performance deficits in schizophrenia are due to a degradation in the internal representation required as context for processing stimuli. In a state of reduced prefrontal dopaminergic activity noise interferes with the ability of the system to maintain a representation of task-relevant information. In the framework we presented this comes down to the situation depicted in figure 5 (bottom) where the system is liable to transitions to other attractors due to small internal or external perturbations of the system. The resulting inability of the system to maintain a stable attractor state over time shows itself at a psychological level as the observed disorders of working memory. More generally, this will lead to an uncontrolled spread of activation and an increase of spontaneous neuronal activity, hence, to a decreased signal-to-noise ratio [34]. Similar results have been described in a neural network simulation performed by Peled & Geva [36].

More recent insights have focused on the differential effects of D1- and D2- dopamine receptor stimulation in the prefrontal cortex [34], [37], [38]. Durstewitz [38] argues, partly based on neural network simulations [39], [40], that D1-stimulation could increase the energy barrier between different network states, making it harder to switch from one state to the other. Due to this, active memory states become more robust to distractors and interference. In dynamical terms, D1-induced changes come down to a deepening and widening of the basins of attraction of prefrontal cortex attractor states (figure 5, top).

The combined effects of D1- and D2-receptor stimulation cause dopamine to drive neural networks through a sequence of phases with opposing characteristics. In the case of prefrontal cortex this may consist of an initial phase where basins of attraction are flattened out (a D2-dominated situation making networks highly susceptible to newly incoming information –see figure 5, bottom), and a late phase (D1-dominated) where network activity is focused on a few relevant states (figure 5, top). Thus, this mechanism is used to ‘protect’ task relevant information against the interfering, and cumulative effects of noise over time [36].

### The metaphor as part of a broader information processing concept

The terms we used are derived from the theory on complex dynamical systems. In this concept the brain is seen as a system that is in constant interaction with its environment, possessing a great number of preferential states in which the system sequentially relaxes [15], [41]. These preferential states are attractors of the systems' dynamics. They are the result of the physical structure of the system and past experiences which have influenced the system. The outside world influences the present situation of the system persistently, causing the system to temporarily relax in a state most fit to this ongoing process of mutual influence.

Homeostatic mechanisms produce stability as well as phase transitions, making the healthy system adaptive to its environment. Psychopathology arises when the system becomes either too stable or too unstable, whether by (acute or chronic) external influences or by limitations in the system itself. In most pathological circumstances, there will be a constant interaction between the system and the environment, resulting in a more or less pathological equilibrium.

It has been suggested to name these aspects of neural functioning which focuses on the system properties of neural ensembles *neurodynamics* [12], [15], [42], [43].

Our approach has been to simplify and visualize these processes, in order to produce an intuitive understanding.

## Discussion

Ideally, medical thinking depends on the understanding of both physical reality and the availability of a metaconcept describing reality in a more abstract way. For instance, in cancer research, the metaconcept is ‘regulation of cell proliferation’, with ‘misregulation’ as its associated pathological state [44]. The physical reality is the way in which the bio-physical apparatus of genes and proteins perform regulation of cell proliferation.

The line of reasoning introduced in this paper tries to approach this situation. The metaconcept defined is based on information processing theory (namely, information processing with attractors [45], [46]), the neurobiological process described (pruning of the dendritic tree) is functionally related with the metaconcept, (pruning optimizes information processing), pathology is understood as arising from disturbances of this neurobiological process (excessive pruning) and the associated pathological phenomena are expressed in terms of the metaconceptual framework (emergence of pathological attractors). Moreover, neuromodulatory influences (dopamine) can be described in terms of the metaconcepts as well.

In psychiatry, there is a large conceptual gap between empirical research and theory which -especially in brain research- must be bridged by an interdisciplinary formal language [47]. In this respect, ‘systems science’ or ‘computational neuroscience’ offers a conceptual and methodological basis for integrating the various data within a sophisticated system framework.

In this context, we think that some ‘metaphorization’ of psychological categories into cybernetic language might be useful to bridge this gap.

Throughout history, the working of the brain has been compared to that of a clock-work, a steam engine and, most recently, to a digital computer or a hologram [48], [49]. The metaphor presented in this article is derived from dynamical systems theory and is a visual representation of the mathematical concepts underlining this theory.

We expect that the conceptual framework and metaphor described in this article offers a tool to understand psychiatric phenomena from a systems perspective. At the same time, we are dealing with a line of thought which is related with the actual behavior of neural systems, so the terminology used, though of an abstract nature, has a close relationship with the processes in the brain. Moreover, it facilitates to appreciate relationships between related phenomena in different fields of science, describing properties and phenomena of neural systems as more specific instances of general principles underlying behavior of all kinds of complex dynamical systems. It therefore satisfies the scientific endeavour to recognize specific behavior (like the behavior of neural sytems) as instances of more general behavior (like the behavior of complex systems) and to construct a conceptual framework describing this general behavior.

Finally, it should be borne in mind that theories and models need not be ‘explanations’ of observed phenomena but can also be useful as exploratory ‘heuristics’ [47]. The presented model might serve this purpose.

## Methods

This is an essay-like study aimed to contruct visualized concepts stemming from the theory on dynamical complex systems to be used as a ‘visual metaphor’ to bridge the mental- and molecular-biological domains in schizophrenia.

The background for this study has been formed by a PubMed search with the terms (‘neural network’ OR ‘connectionism’ OR ‘parallel distributed processing’ OR ‘nonlinear systems’ OR ‘complex systems’) AND (‘schizophren*’ OR ‘psychosis’ OR ‘psychotic’ OR ‘psychiatr*’).

The general concept of a (visual) metaphor is inspired by the work of Globus & Arpaia [6], Draaisma [48], [49], and Ouzounis & Maziere [50].

To introduce the sought-after metaphor we first describe a computer model of neural information processing. We then show how the information processing in this model can be visualized, using concepts from the theory on complex systems. Finally, we use the visual metaphor to describe disturbed information processing and dopamine neuromodulation in schizophrenia.
